# Problems with two recent Petri net analyses of Neanderthal adhesive technology

**DOI:** 10.1038/s41598-024-60793-1

**Published:** 2024-05-07

**Authors:** Patrick Schmidt, Claudio Tennie

**Affiliations:** 1 Department of Early Prehistory and Quaternary Ecology, Eberhard Karls University of Tübingen, Tübingen, Germany; 2 Applied Mineralogy, Department of Geosciences, Eberhard Karls University of Tübingen, Tübingen, Germany

**Keywords:** Archaeology, Social anthropology

**arising from**: P. R. B. Kozowyk et al.; *Scientific Reports* 10.1038/s41598-023-41963-z (2023).

**arising from**: S. Fajardo et al.; *Scientific Reports* 10.1038/s41598-023-42078-1 (2023).

## Introduction

Birch tar making by Neanderthals was one of the first transformative processes in human history. It has implications for our understanding of the cognitive capacity and cultures of early humans. However, it has been shown that birch tar finds cannot be standalone proxies for these processes. This is so because birch tar may be the result of condensation from burning bark onto flat stone surfaces^1^, which can happen even in fortuitous accidents. Thus, the tar making techniques actually used must be investigated and their specific implications for cognition and culture must be properly understood. It is therefore generally welcome that Kozowyk et al.^2^ intend an interpretation of the condensation method’s implications. They do so for a specific application: the case where three cobbles act as condensation centers. Using the same approach, Fajardo et al.^3^ interpret the meaning of several birch tar making techniques. There are several problems with their approach, and here we will address three of them.

## The need of “scaling up” the condensation method

The argument that several stones can be used simultaneously in the condensation method was initially brought forward^4^ merely to show that efficiency estimations (as in Niekus et al.^5^) are not straightforward. It was not claimed that the amount of cobbles tested in that study (three) were actually used—and in this way—by Neanderthals. And indeed, it has recently been shown^6^ that Neanderthals made birch tar with an elaborate underground technique that was most likely the improvement of an earlier aboveground technique. Thus, a specific claim that the condensation method “likely needed to be scaled up”^2^ is not rooted in archaeological data.

## Degree of freedom and subjectivity in Petri nets

Kozowyk et al.^2^ and Fajardo et al.^3^ argue for process complexity based on Petri nets, which are vector addition systems putting into relation “places” (conditions or locations) and “transitions” (things that are done or that happen). Petri nets are normally used for analysing hardware, software or business processes. Such nets can be generated using a semi-automated software, suggesting a certain degree of objectivity to an analyst. However, especially in archaeology, the choice of “places”, “transitions” and “arcs” (the constituting elements in Petri nets), can easily become arbitrary. To describe a process like tar making, these elements must largely be invented by the analyst. This is so because there are only indirect ways of knowing what people actually did while conducting processes. Examples from both papers^2,3^ illustrating the problematic nature of this approach are for example elements called “place rocks” [on the ground], “bark extinguishes” or “start condensation”. Regardless of whether they describe something one must do or something that just happens, these elements count towards the complexity of tar making in Petri nets^2^ (Fig. 4). One might argue that there is a worrying inbuilt degree of freedom here. Analysts can choose other elements (additionally or instead), such as *positioning rock relative to the wind*, *flattening the soil surface* before placing the cobble, or processes such as *cobbles are progressively getting hot*, *fire spreads to the remainder of the bark*. Because there is no objective way to constrain and define these steps, the choice of elements reflects the analyst’s subjective interpretation. Even if chosen steps are encoded using the syntax of Petri nets (or anything similar) the number of places, transition, and arcs that are eventually counted to make comparative statements (Fig. 4^2^; Table 1^3^) is strongly influenced by the analyst’s subjective choices, an approach with predictable negative effects for reliability measures^7^.

## Risk and decisions in three-cobble-condensation

Kozowyk et al.^2^ interpret their findings as follows. They claim that using three cobbles instead of one requires a massively greater amount of decision-making, attention capacity, working memory and inhibitory control. The reason for this is that, with three cobbles, there is greater risk that a flame could go off (and must be re-ignited on an “external source”^2^) or that tar would “burn away”^2^ from a cobble. Such incidents are explained to constitute failures in the tar making process that need to be anticipated. Based on these assumptions, the Petri net choice of Kozowyk et al.^2^ becomes more complex for the three-cobble-version, in fact ~ 120 times more complex (the metrics are called reachability graphs and ECyM). This is put in the context of 262 other Petri nets, made for hypothetical modern business decision processes, which are almost all described as simpler^8^ (the adequacy of comparing ancient tar making with business processes remains unclear). Relatedly, some complex tasks are suggested to exceed the cognitive capacities of individual Neanderthals^2^, and their discussion paragraph suggests that the condensation method using three cobbles is one such task. Whether this is a realistic assumption or not can be tested experimentally. For example, from supplementary video 2 in Blessing and Schmidt^4^, showing one of us (PS) conducting the three-cobble-condensation method, it can be seen that: all bark rolls are lit on other burning rolls (instead of an “external source”^2^); cobbles are scraped 10 times, their order changes; no tar is burning off any of the cobbles; eight times, flames go off and are reignited; in most cases, they are reignited by adding new burning bark to the same cobble so that flames jump over; during five (half) of the scraping processes, there is one cobble where no bark is burning; in the end, 0.3 g of birch tar are produced^4^. Hence, there is no risk that tar burns off (we have never witnessed such an incident in any of our condensation method experiments^1,4,9–11^). There is also no specific order of steps that must be followed during the process. Flames going off while another cobble is scraped^2^ does not compromise the success of the method. In other words, it cannot be considered an error and needs no anticipation. Thus, Kozowyk et al.’s^2^ assumptions about risk are erroneous. They are based on misperceptions of the actual process space as well as on mistakenly equaling a slightly imperfect execution with a failed one. And indeed, several condensation experiments based on the same sequence but conducted by other operators were also successful^4,10^. Two times, these experiments included the first attempt of an operator to conduct the method. Both first tries were immediately successful^4,10^, highlighting that there was no (or little) learning curve. From the memory of one of us (PS), none of the operators experienced difficulties or reported stress during the experiments. Thus, at least for modern *Homo sapiens*, our observations appear to falsify Kozowyk et al.’s^2^ hypothesis, which proposed that the three-cobble-condensation method represents a particularly high cognitive load. Whether this is also true for Neanderthals cannot be directly tested, but it appears likely.

## Conclusion

Artefacts that document early transformative technology, such as ancient adhesives^5^ or heat-treated rocks^12^, are among our best material evidences that help understand the cognitive and cultural evolution of early humans. Several ways of interpreting archaeological artefacts and techniques have been proposed^13–15^, providing a tool-kit archaeologists can use to pinpoint the advent of physical and cultural changes in human history. Interpreting the step-by-step complexity of ancient processes, even using methods like Petri nets^2,3^, is among the least promising approaches because it includes the analyst’s preconceptions and biases, while not relying on reproducible or testable predictions.
